# Validation of Material Algorithms for Femur Remodelling Using Medical Image Data

**DOI:** 10.1155/2017/5932545

**Published:** 2017-12-26

**Authors:** Shitong Luo, Xingquan Shen, Xin Bai, Jing Bai, Jianning Han, Yu Shang

**Affiliations:** ^1^North University of China, No. 3 Xueyuan Road, Taiyuan 030051, China; ^2^Tsinghua University, No. 30 Shuangqing Road, Beijing 100084, China

## Abstract

The aim of this study is the utilization of human medical CT images to quantitatively evaluate two sorts of “error-driven” material algorithms, that is, the isotropic and orthotropic algorithms, for bone remodelling. The bone remodelling simulations were implemented by a combination of the finite element (FE) method and the material algorithms, in which the bone material properties and element axes are determined by both loading amplitudes and daily cycles with different weight factor. The simulation results showed that both algorithms produced realistic distribution in bone amount, when compared with the standard from CT data. Moreover, the simulated L-T ratios (the ratio of longitude modulus to transverse modulus) by the orthotropic algorithm were close to the reported results. This study suggests a role for “error-driven” algorithm in bone material prediction in abnormal mechanical environment and holds promise for optimizing implant design as well as developing countermeasures against bone loss due to weightlessness. Furthermore, the quantified methods used in this study can enhance bone remodelling model by optimizing model parameters to gap the discrepancy between the simulation and real data.

## 1. Introduction

The effects of mechanical loadings on bone amount and skeleton structure are widely recognized. The osteocytes embedded in mineralized bone matrix sense the mechanical stimulus and modulate the activities of osteoblasts and osteoclasts, which, respectively, are responsible for bone formation and absorption [1]. For certain people who are in an abnormal mechanical environment, such as those having undergone arthroplasty, the stress and strain around their hip or knee joints are different from those of normal persons [2]. This is known as “stress shielding,” and it may lead to bone resorption around femoral stems [3]. Another example is that of astronauts in space, where bone loss may occur due to the microgravity environment [4]. A relationship exists between mechanical stimuli and the density or strength of bone which, consequently, affect skeletal function. Thus, it is necessary to investigate the alteration of bone quality due to changes in the mechanical environment. Although the exact mechanism on how the bone responds to mechanical loading is unclear, plenty of models (i.e., bone material algorithms) exist describing internal bone formation and absorption under certain mechanical loads. For example, the “reaction-diffusion” model [5] is based on the osteocyte mechano-sensory network consisting of activator and inhibitor for bone formation activity. The bone growth or absorption is determined by the concentrations of activator and inhibitor, which is adjusted by the von Mises stress according to reaction-diffusion equations. Another model is based on “damage-repair theory” [6–8], in which bone absorption is seen as the “damage” process leading to an increase of porosity; while the bone formation is seen as the “repair” process that results in a decrease of porosity, the damage-repair process is influenced by the mechanical stimulus via a defined “damage-repair” tensor. Both models, however, are not well validated or widely accepted at present; moreover, the parameters involved are not easy to obtain experimentally or empirically. The dominant model for the description of bone remodelling is the “error-driven” algorithm [9–16], in which the bone material is adjusted by the error between current mechanical stimulus and a reference value. The stress, strain, or strain energy density usually serve as the mechanical stimulus. The key hypothesis for this algorithm is that higher mechanical stimuli lead to an increase in the amount of local bone, whereas lower mechanical stimuli lead to a decrease. Under certain mechanical loads, the bone density and stiffness will repeatedly change until the mechanical stimulus at every location reaches its preset value, which is known as “reference stimulus” [17]. If the mechanical loads change, the bone material will redistribute so as to attain the new equilibrium. The “error-driven” bone material algorithm has been developed assuming bone is either an isotropic [4, 14, 18–20] or orthotropic material [16, 21, 22]. This algorithm was also used by various groups to predict bone remodelling following total hip arthroplasty [15, 23] and dental implants [12, 16], as well as evaluating bone loss prevention treatments [24]. The difference (i.e., “error”) of the mechanical stimulus (e.g., stress, strain, and strain energy density) under normal and abnormal (e.g., total hip arthroplasty) mechanical loads is chosen as the “driving force” to alter the bone mass or material properties [23]. These prediction studies pave the way for optimizing implant designs to preserve bone mass and developing countermeasures against bone loss due to weightlessness. The bone material algorithms need to be validated since the accuracy of model is critical for predicting the change of bone quality under abnormal mechanical environments, and quantified evaluation criterions are required for the algorithm validation. To the best of our knowledge, however, there is no quantified method for algorithm evaluation. Instead, most of the algorithms are only qualitatively validated. For example, the distribution characteristics of simulated bone mass in typical regions of the femur, such as femoral head and neck, Ward's triangle, and greater trochanter, were used to check the consistence with the observed bone mass distribution in real anatomical femur [21, 25]. For orthotropic algorithm, the stiffest direction of Young's modulus is used as another criterion for checking the trabecular alignment [21]. These qualitative methods provide a rough comparison between the simulated and real bone quality, but have no capability to qualify the difference in individual locations. So, the quantified method is required to evaluate, calibrate, and modify the bone material algorithm for gapping the differences between the simulated and real results. Computed tomography (CT) is one of the principal medical modalities for imaging bone structure due to the high contrast between the bone and surrounding soft tissue. The CT Hounsfield unit (HU) has an approximately linear relationship with bone apparent density [26–28], thus allowing for quantifying the real bone amount to evaluate bone remodelling simulation.

This study used CT data for quantitative validation of the two types of “error-driven” algorithm: isotropic algorithm, which considers bone as an isotropic material, and orthotropic algorithm, which considers bone as an orthotropic material. The isotropic algorithm was chosen for validation because it has been used by numerous groups for bone material prediction [4, 14, 18–20]. To eliminate the limitation of the isotropic assumption, Miller et al. [21] proposed an orthotropic algorithm describing the trabecular alignment and material orientation. As a simplified two-dimensional (2D) model, Miller's algorithm uses stress to calculate the material axes, and only a single load case is considered for the determination of each element axes. We extended Miller's 2D model to a three-dimensional (3D) model in our previous research [22]. In addition, the material axes in our algorithm are determined by both daily load cycles and the stress under multiple load cases, making it more complex and realistic. We verified that this algorithm produces more realistic bone distribution than Miller's algorithm [22], thus choosing it as the orthotropic algorithm for validation in this study.

The bone remodelling simulation under multiloading conditions was implemented via iterative calculations. The Young's modulus of bone changed according to the isotropic or orthotropic algorithm, in which a strain-based variable was defined and chosen as the mechanical stimulus. The numerical computation was implemented by combining the bone material algorithm with the finite element (FE) method. The FE model was established based on the CT data of the human femur. Using both algorithms, the ultimate stiffness and density of bone that adapt to the mechanical environment were obtained after the iteration came to convergence. Bone density is chosen as the most important parameter for evaluating simulation results because its distribution follows bone material theory that bone grows in the areas with high loadings, while bone absorbs in the areas with low loadings. The correlation coefficient and mean error between the real densities and simulated densities were used for algorithm evaluations. Besides, for orthotropic algorithm, the trabecular orientation and the ratio of longitude modulus to transverse modulus (L-T ratio) were obtained and used to evaluate simulation results since they characterize the structural features of bone in response to loadings. The results showed that the “error-driven” algorithm was efficient in describing the bone remodelling process. Furthermore, this study provides a general method for quantified evaluation, calibration, and improvement of bone remodelling models by introducing the actual data into the algorithms.

## 2. Material and Methods

### 2.1. FE Model

A 3D geometrical model was reconstructed from the male CT data set obtained from the Visible Human Project (VHP) of the National Library of Medicine (NLM, USA). As the first data set of human anatomical models, the VHP data are considered as a standard and used for many biomedical applications, such as the virtual surgical planning and computerized visualization. The male CT data is also widely used in field of Biomechanics, such as the studies reported in [10, 22, 29]. Following the procedures for image processing and image reconstructions reported by us and others [11, 29, 30], the 3D geometrical model was transferred to a commercial FE software MARC (MSC, USA). The automatic meshing function in Mentat (MSC, USA) was used for generating high-quality eight-node hexahedron elements within the femur, with element size of 3.0 mm. The number of elements and nodes was 24,441 and 29,161, respectively. The local Cartesian coordinate system of the femur was defined as (*x*) medial-lateral, (*y*) anterior-posterior, and (*z*) superior-inferior. For the purpose of density assignment, the centric coordinate of each element was calculated by averaging the coordinates of its eight nodes. The nearest CT sampling point to the centric coordinate was chosen, and the HU of sampling point was assigned to this element. The marrow was neglected in this study. The HU below 150 was assigned to be 150, and the HU above 1500 was assigned to be 1500. Due to the linear relationship between the bone apparent density and HU, the real density was denoted as [28, 29]

(1) $$\begin{array}{c} \rho \;\left(g/{cm}^{3}\right)=\frac{2HU}{1500}. \end{array}$$

The cortical bone was assumed in the case of bone density from 0.946 to 2.0 g/cm^3^, and the cancellous bone was assumed in the case of bone density from 0.2 to 0.945 g/cm^3^.

In order to compare with the simulated bone density, the procedures depicted in [28, 29] were utilized by us to calculate the HU value of CT (as well as the real bone density via (1)) for each FE element. Briefly, all voxels contained in a given FE element are determined through the coordinate range of element vertexes. Then, the HU value is summed over all voxels to obtain the HU value (as well as the bone density via (1)) of the FE element, which is comparable to the simulated values at the same scale.

At the start of bone remodelling simulation, the whole femur was assumed to be homogeneous and isotropic with *E* = 1737 MPa and *ν* = 0.3. The distal femur was constrained in all directions. Three different load cases, representing hip-joint and abduct-muscle forces, were applied to the femoral head and the greater trochanter, respectively, which were the same as those adopted by Doblaré and Garcia [6]. Both cortical bone and the cancellous bone were assumed to remain isotropic when the isotropic algorithm was adopted, while it became orthotropic when the orthotropic algorithm was adopted.

### 2.2. Material Algorithm

The orthotropic material algorithm includes two steps: the determination of orthotropic axes and the bone stiffness modification. This algorithm is based on the 2D algorithm initiated by Miller et al. [21]. We improved this algorithm and extended it for 3D application [22]. The key difference of Miller's 2D algorithm and our 3D algorithm is the method for determination of orthotropic axes. In 2D model, only the stress under a single load case is used to calculate the material axes of each element and the load cycles are excluded for determination of element axes. While in 3D model, the material axes are determined by multiple load cases for any element and both the stress and load cycles are considered for axes determination with different weight factor. For each element, three local material axes are determined step by step. First, three principal stresses of each element under each load case are rearranged according to their magnitudes:

(2) $$\begin{array}{c} \left|\sigma_{1\left(i,j\right)}\right|\geq \left|\sigma_{2\left(i,j\right)}\right|\geq \left|\sigma_{3\left(i,j\right)}\right|. \end{array}$$

The direction of *k*th axis in *i*th element under *j*th load case is denoted as a unit vector ${\vec{g}}_{k\left(i,j\right)}.$

Then, a new vector is defined as

(3) $$\begin{array}{c} {\vec{f}}_{k\left(i,j\right)}={\left(n_{j}\right)}^{1/m}\left|\sigma_{k\left(i,j\right)}\right|{\vec{g}}_{k\left(i,j\right)}\;\left(k=1,2,3\right), \end{array}$$

where *m* is an empirical constant. The larger *m* value indicates that the principal stress weighs more heavily than the load cycles. The *n*_*j*_ is the cycles of *j*th load case per day. The first direction of the local material axes is determined by the formula:

(4) $$\begin{array}{c} {\vec{e}}_{1\left(i\right)}=\frac{\sum_{j}{\vec{f}}_{1\left(i,j\right)}}{\left|\sum_{j}{\vec{f}}_{1\left(i,j\right)}\right|}. \end{array}$$

Next, we calculate the projection of ${\vec{f}}_{2\left(i,j\right)}$ in the plane perpendicular to ${\vec{e}}_{1\left(i\right)},$ that is,

(5) $$\begin{array}{c} {\vec{pf}}_{2\left(i,j\right)}={\vec{f}}_{2\left(i,j\right)}-\left({\vec{f}}_{2\left(i,j\right)}\cdot {\vec{e}}_{1\left(i\right)}\right){\vec{e}}_{1\left(i\right)}. \end{array}$$

Similarly, the second direction is determined by the formula:

(6) $$\begin{array}{c} {\vec{e}}_{2\left(i\right)}=\sum_{j}{\vec{pf}}_{2\left(i,j\right)}\left|\sum_{j}{\vec{pf}}_{2\left(i,j\right)}\right|. \end{array}$$

Finally, the third direction is

(7) $$\begin{array}{c} {\vec{e}}_{3\left(i\right)}={\vec{e}}_{1\left(i\right)}\times {\vec{e}}_{2\left(i\right)}. \end{array}$$

Thus, the local material axes are determined and every two axes of them are perpendicular to each other.

After the determination of orthotropic axes, the Young's moduli of bone change along each axis according to the “error-driven” algorithm [21, 22]:

(8) $$\begin{array}{c} \frac{{dE}_{k\left(i\right)}}{dt}=\left\{\begin{array}{l} B\left[\Psi_{k\left(i\right)}-\Psi_{0}\left(1+s\right)\right]\;\text{if }\Psi_{k\left(i\right)}\geq \Psi_{0}\left(1+s\right),\\ 0\text{ if }\Psi_{0}\left(1-s\right)<\Psi_{k\left(i\right)}<\Psi_{0}\left(1+s\right),\\ B\left[\Psi_{k\left(i\right)}-\Psi_{0}\left(1-s\right)\right]\;\text{if }\Psi_{k\left(i\right)}\leq \Psi_{0}\left(1-s\right), \end{array}\right. \end{array}$$

where *E*_*k*(*i*)_ represents the Young's modulus in *k*th (*k* = 1, 2, 3) principal axis of the *i*th element. *B* is the remodelling coefficient controlling the increment of Young's modulus in each iteration step [18]. Ψ_0_ is the reference stimulus. The idea of “lazy zone” [31] is employed in this algorithm, and *s* is the width of the lazy zone. The mechanical stimulus is

(9) $$\begin{array}{c} \Psi_{k\left(i\right)}=\frac{1}{E_{k\left(i\right)}}{\left(\sum_{j}n_{j}\left|\sigma_{k\left(i,j\right)}^{m}\right|\right)}^{1/m}. \end{array}$$

It is a strain-based variable. *m* has the same meaning as that in (3). The shear moduli of bone also change according to the formula:

(10) $$\begin{array}{c} G_{ij}=\frac{\left(E_{i}+E_{j}\right)}{4\left(1+\nu \right)},\;\left(ij=12,23,31\right). \end{array}$$

The Poisson's ratio *ν* was assumed constant during the iterative calculation.

No need to determine the material axes for isotropic algorithm, and the corresponding change of Young's moduli is as follows:

(11) $$\begin{array}{c} \frac{{dE}_{\left(i\right)}}{dt}=\left\{\begin{array}{l} B\left[\Psi_{\left(i\right)}-\Psi_{0}\left(1+s\right)\right]\;\text{if }\Psi_{\left(i\right)}\geq \Psi_{0}\left(1+s\right),\\ 0\;\text{if }\Psi_{0}\left(1-s\right)<\Psi_{\left(i\right)}<\Psi_{0}\left(1+s\right),\\ B\left[\Psi_{\left(i\right)}-\Psi_{0}\left(1-s\right)\right]\;\text{if }\Psi_{\left(i\right)}\leq \Psi_{0}\left(1-s\right), \end{array}\right.\\ \Psi_{\left(i\right)}=\frac{1}{E_{\left(i\right)}}{\left(\underset{j}{\sum }n_{j}\left|\sigma_{\left(i,j\right)}^{m}\right|\right)}^{1/m},\\ \;\left|\sigma_{\left(i,j\right)}\right|=\frac{\left(\left|\sigma_{1\left(i,j\right)}\right|+\left|\sigma_{2\left(i,j\right)}\right|+\left|\sigma_{3\left(i,j\right)}\right|\right)}{3}. \end{array}$$

The bone density is calculated by the following formula [32]:

(12) $$\begin{array}{c} E\left(Mpa\right)=\left\{\begin{array}{c} 1904\rho^{1.64},\;\rho <0.946\;\left({g/cm}^{3}\right),\\ 2065\rho^{3.09},\;\rho \geq 0.946\;\left({g/cm}^{3}\right). \end{array}\right. \end{array}$$

For orthotropic algorithm, *E* is the average of the Young's moduli in three principal axes. If the mean density variation is less than 0.001 g/cm^3^, the iteration stops, indicating that the steady structure of the femur is attained and it adapts to the applied mechanical loads.

### 2.3. Evaluation

The real density was defined as the bone density acquired from the CT data according to (1), and the simulated density was defined as the bone density simulated by the isotropic or orthotropic algorithm when iteration reaches convergence.

One criterion for algorithm evaluation is the correlation coefficient between the real and simulated density over the whole femur. The larger correlation coefficient means that the simulated density distribution coincided more with the real density distribution. Another evaluation criterion is the mean error between the real and simulated density for all the elements. It is defined as follows:

(13) $$\begin{array}{c} \Delta \rho^{mean}=\frac{\sum_{i}\left|\rho_{i}^{simu}-\rho_{i}^{real}\right|V_{i}}{\sum_{i}V_{i}}, \end{array}$$

where Δ*ρ*^mean^ is the mean error of bone density, *ρ*_*i*_^simu^ and *ρ*_*i*_^real^ are the simulated and real density in *i*th element, respectively, and *V*_*i*_ is the volume of *i*th element. The smaller mean error indicates that the simulated bone density is closer to the real bone density. In addition, the mean density in several local regions of the proximal femur, that is, the superolateral, inferomedial, and diaphyseal region, is also calculated and used to investigate the bone amount difference among the subregions of the femur.

As for the orthotropic algorithm, the L-T ratio is used to describe the difference between the vertical and horizontal Young's modulus in local regions of the proximal femur. The moduli in *x*, *y*, and *z* directions are derived from the stiffness matrix in the FE analysis, and the L-T ratio is defined as 2*E*_*zz*_/(*E*_*xx*_ + *E*_*yy*_). Besides that, the trabecular alignment in coronal section of the proximal femur is also displayed.

## 3. Results

All the parameters except Ψ_0_ remained constant with *B* = 50000, *m* = 4, and *s* = 0.1 in this simulation study. A series of discrete values of the reference stimulus Ψ_0_ were used in order to find the most suitable value with which the realistic density distribution is achieved. A Python script was designed to control the iteration of FE analysis and update element material properties. Then the updated material properties were input into MARC via FORTRAN user subroutine for the next step of FE analysis. The Gauss integral and Gauss-Seidel iterative method were used to generate and solve the stiffness matrix in FE calculation, respectively. When the solution difference between two adjacent iterations is smaller than the tolerance criterion of 0.001, the calculation is considered to reach convergence. To avoid the “check-board” effects, the element stress was obtained by averaging the stress values of eight nodes within the element, as did by others [6, 33]. Desired calculation stability was achieved since the singularity ratio is larger than 10^−4^ for all FE calculations. It took 53 and 46 steps, respectively, for the isotropic and orthotropic iterations to reach convergence.

### 3.1. Validation of Distribution in Bone Amount

**Figure 1:**
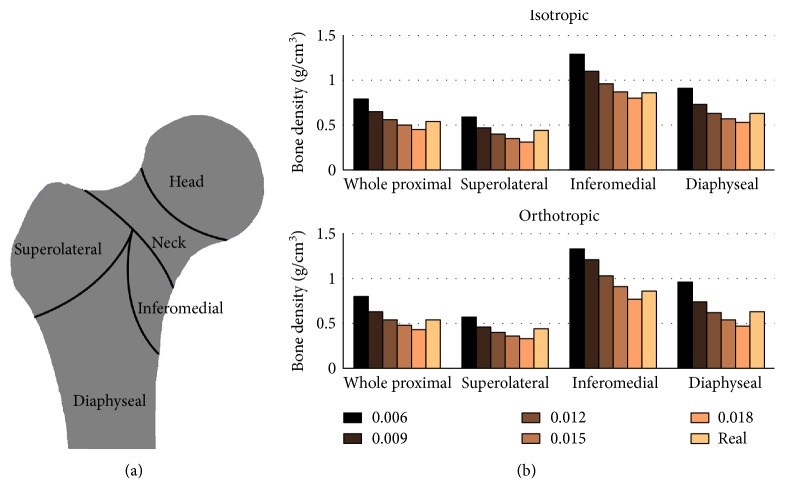
(a) The local regions in the proximal femur, including the superolateral, inferomedial, diaphyseal, and femur head and neck. (b) The real and simulated bone densities (g/cm^3^) in the local regions of the proximal femur. The real densities are obtained from CT data. The simulated densities are calculated by the isotropic and orthotropic material algorithms, respectively.

The values of real and simulated mean densities in four local regions of the proximal femur were exhibited in Figure 1. Obviously, all local densities decreased with the increasing Ψ_0_. For any Ψ_0_, the inferomedial, diaphyseal, whole proximal, and superolateral regions were the regions with the density from the largest to the smallest, which coincided exactly with the magnitude sequence of real density in these regions. The simulated local density achieved the closest value to the real density when Ψ_0_ was 0.012. In the superolateral, inferomedial, diaphyseal, and whole proximal regions, the differences of local density were 9.1%, 11.6%, 0.1%, and 3.7%, respectively, with the isotropic algorithm, and 9.1%, 19.8%, 1.6%, and 0.1%, respectively, with the orthotropic algorithm.

**Figure 2:**
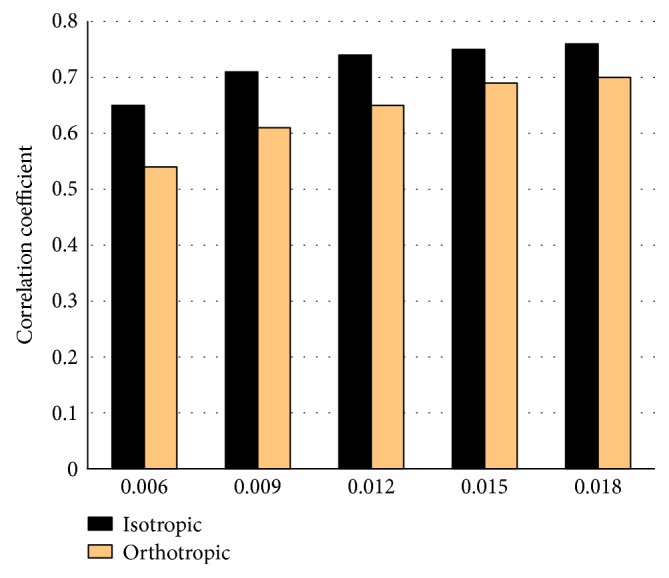
The correlation coefficients between the real bone densities and those simulated by the isotropic and orthotropic algorithms. Five values of reference stimulus Ψ_0_ (0.006~0.018) are used for simulation, respectively.

The correlation coefficients between the real and simulated densities in the whole femur were displayed in Figure 2. They increased with the increasing reference stimulus Ψ_0_ at the beginning, then reached maximums. For the same Ψ_0_, the correlation coefficient was a little larger with the isotropic algorithm than orthotropic algorithm, and the maximum was 0.76 and 0.70, respectively.

**Figure 3:**
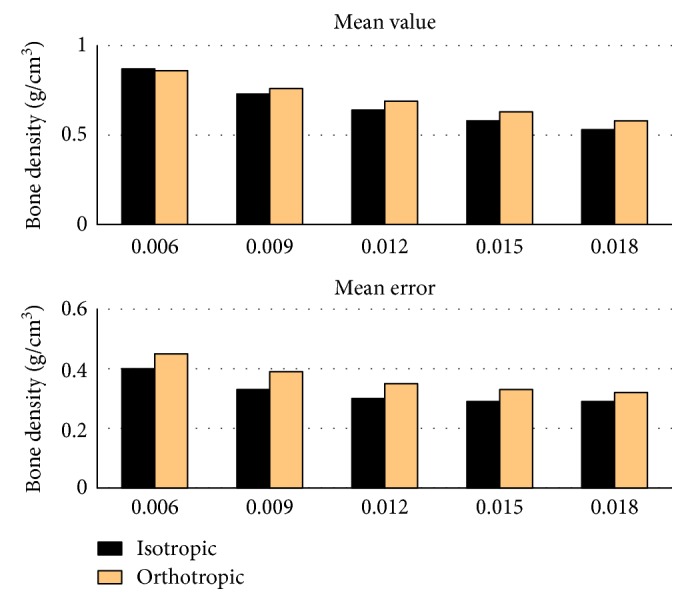
The mean value and mean error of the bone densities simulated by the isotropic and orthotropic algorithms. Five values of reference stimulus Ψ_0_ (0.006~0.018) are used for simulation, respectively.

Figure 3 exhibits the values of simulated mean densities and mean errors relative to the real densities. The simulated mean density decreased with the increasing Ψ_0_, while the mean error decreased at the beginning and then reached minimums. For the same Ψ_0_, both mean density and mean error were slightly smaller with the isotropic algorithm than orthotropic algorithm. The minimal errors were 0.29 and 0.32 g/cm^3^, respectively.

**Figure 4:**
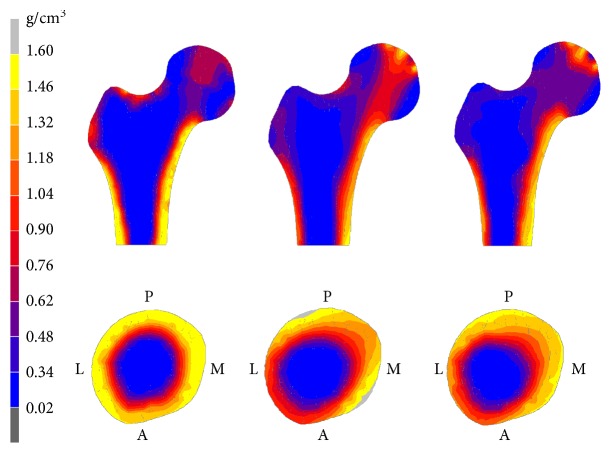
The bone density distribution in coronal and transverse section of the proximal femur. The densities obtained from the CT data, simulation by the isotropic algorithm, and simulation by the orthotropic algorithm were shown from left to right. The upper row is the density distribution in coronal section. The lower row is the density distribution in transverse section. L: lateral; M: medial; A: anterior; P: posterior. The value of Ψ_0_ used in both algorithms was 0.012.

Figure 4 shows the real and simulated density distributions in the proximal femur when Ψ_0_ was 0.012. The medial and lateral diaphysis, femoral head, and the lesser trochanter showed larger density than the greater trochanter and the inner diaphysis, which was in agreement with the distribution characteristic of real density. The difference was that the real bone amount was highly concentrated and the transition from high-density region to low-density region was sharper, whereas the simulated bone amount was more uniform and the transition was smoother.

### 3.2. Validation of Bone Mechanical Properties

**Figure 5:**
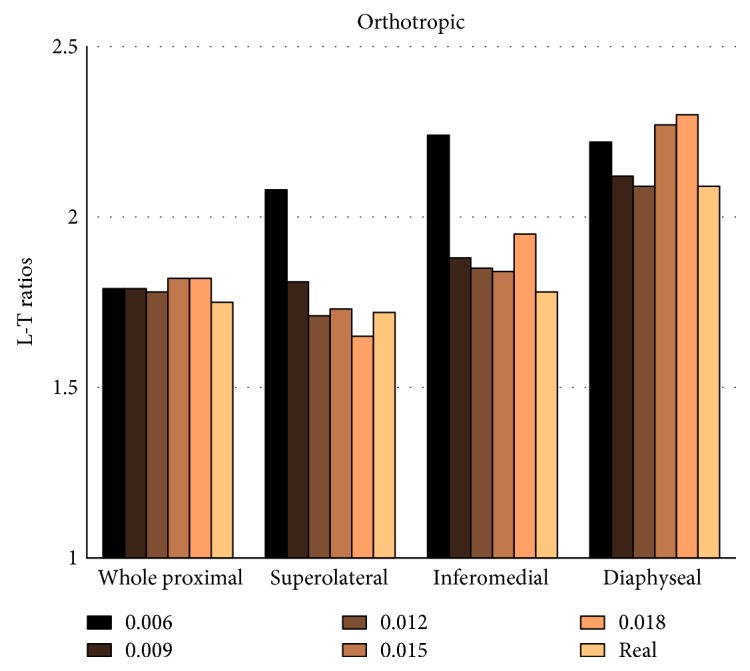
The simulated L-T ratios in the local regions of the proximal femur, which were calculated by the orthotropic material algorithm. The real L-T ratios derived from literature [34] are also illustrated for comparison.

Figure 5 exhibits the L-T ratio simulated by orthotropic algorithm and derived from literature in the local regions of the proximal femur. Little relationship was found between the L-T ratio and reference stimulus. The L-T ratios were larger in the diaphyseal and inferomedial region than the superolateral region. They achieved the closest values to the reported results [34] when Ψ_0_ was 0.012. The difference was 0.58%, 3.93%, 0.1%, and 1.71% in the superolateral, inferomedial, diaphyseal, and the whole proximal regions, respectively.

**Figure 6:**
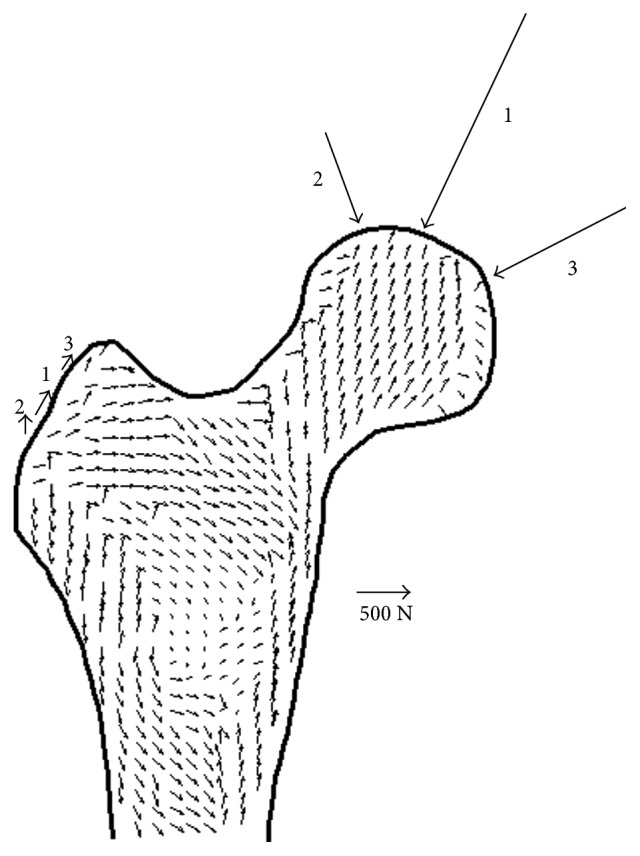
The projections of the element principal axes in coronal section. The orthotropic axes of each element were determined by three load cases according to the orthotropic algorithm, and only the first principal axis is shown in the figure. The trabecular alignment is also displayed since the first principal axis is the most stiffness axis, which aligns with the trabecula. The magnitudes and the directions of forces applied to the proximal femur in load case 1, 2, and 3 are also shown in the figure, and the magnitude of scale arrow is 500 N.

The trabecular alignment simulated by orthotropic algorithm is shown in Figure 6. The arrows represented the projections of the first principal axes in coronal section, and they also denoted the trabecular orientations because the most stiffness direction aligns with the trabecula. In addition, three load cases, representing the primary forces on the femoral head and greater trochanter, are also displayed in coronal section. The arrow length represented the force magnitude, and the arrow direction represented the force direction projected in coronal section. Clearly, the trabecular orientations aligned with the hip joint loading directions in the femoral head and aligned with the muscle tension directions in the greater trochanter. While in the diaphysis, most trabeculae were arrayed vertically due to the ground gravity. These alignment characteristics verified the hypothesis that trabecula matches with the stress trajectory [5, 35, 36].

## 4. Discussion

There are two aims for bone remodelling simulations: to design or validate new model or to predict bone remodelling using existing model. For model validation, the internal bone is assumed as isotropic and homogeneous initially and it will then be changed by mechanical loadings according to a specific remodelling algorithm. For bone remodelling prediction, however, the real bone density and structure are chosen as the initial state, and the error of mechanical stimulus under abnormal loadings (e.g., hip implant and weightlessness) relative to that under normal loadings is chosen as the “driving force” to induce bone remodelling over time.

Two “error-driven” bone material algorithms, that is, isotropic algorithm and orthotropic algorithm, were validated in this study. The mechanical stimulus is a scalar in isotropic algorithm. The equivalent stress, equivalent strain, and strain energy density are often chosen as the candidate stimulus. Turner et al. [36] demonstrated that the average strain is more appropriate to be the mechanical stimulus when compared to the strain energy density. For orthotropic algorithm, however, the mechanical stimulus is an essential vector so as to display the orientation of bone material. Considering the contribution of multiple load cases to bone remodelling, we improved the material algorithm proposed by Miller et al. [21] in our previous study [22] and selected it as the orthotropic algorithm for validation purpose in this study. Correspondingly, the mechanical stimuli in three principal material axes were averaged and used as the mechanical stimulus in isotropic algorithm.

The isotropic algorithm has the advantages of simplicity and less time-consuming in both FE analysis and iterative calculation; thus, it is preferred in the situations where only bone density is the major concern, whereas tissue anisotropy is neglected. For some studies such as bone loss [37, 38], the bone amount loss following hip arthroplasty was solely concerned, since bone amount was measurable and comparable with clinical standards (e.g., DEXA measurement). The disadvantage of isotropic algorithm is obvious, that is, cannot reflect the bone tissue anisotropy, which is critical in various studies such as strength and fracture analysis. Many studies have demonstrated that bone strength is not solely dependent on bone apparent density; microstructure and anisotropy also contribute greatly to bone strength [39]. Thus, in the abovementioned situations, the bone tissue anisotropy has to be taken into account. The orthotropic algorithm outputs multiple variables describing bone tissue anisotropy (e.g. material orientation and Young's moduli in principal axes), therefore allows for introducing additional criteria (e.g., L-T ratio in the present study) along with bone density for algorithm evaluation. Note that orthotropic algorithm requires the determination of orthotropic axes by (2)–(7); it thereby introduces complexity to FE analysis and iterative calculation (see Section 2.2), consequently prolonging the calculating time for bone remodelling simulation. The isotropic algorithm does not need to calculate the material axes due to isotropic assumption; thus, it is easily implemented in FE analysis and can save the calculating time.

The dual energy X-ray absorptiometry (DEXA or DXA) is currently the standard for bone quantity evaluation in clinic and has been used to assess the bone remodelling prediction in the study of postoperative hip implant [40]. However, DEXA only permits surface density measurement, which, although is sufficient in clinical diagnosis, cannot characterize the spatial heterogeneity of bone. Compared with DEXA, the medical CT data not only offer abundant information of bone amount but also can be used to reconstruct 3D bone structure accurately [29].

This study evaluated the isotropic and orthotropic algorithms in describing bone remodelling process. In contrast to previous reports focusing on experiential description of the simulated distribution in bone amount [18, 21], we used medical CT as a standard to quantify the errors of simulated bone amount relative to real bone amount, which allows for a quantified comparison between multiple bone material algorithms. Although some factors, such as the relations between CT density and material modulus [41], as well as the muscle forces [42], may affect the results of mechanical analysis, the pattern of bone remodelling results would not significantly alter.

The validation study showed that the realistic density distribution was attained when an appropriate reference stimulus is chosen for the presented femur model (see Section 3.1): 0.012 is the optimal value for both isotropic and orthotropic algorithm, resulting in relative high correlation coefficient (0.74 and 0.65) and relative small mean error (0.30 and 0.35 g/cm^3^).

This validation study is limited by several factors. First, the “error-driven” algorithm is an apparent method, which focuses on the relationship between the local bone amount/structure and mechanical stimulus, and does not deeply reflect the biology mechanism involved in bone remodelling. Second, this algorithm considers the reference stimulus as non-site-specific [43], that is, the mechanic stimulus at every location attains the same value when iterative calculation reaches equilibrium. The errors will be produced if the reference stimulus is site-specific, or in other words, there are no uniform hypotheses exist. Third, the bone was set as linear elastic material and the marrow was excluded during FE analysis, as most researchers did. That would lead to the errors. Besides, the physiological loadings on the femur are much more complicated than used in this study, where only the hip joint contact forces and abductor force were included in each load case. This set of simplified boundary conditions is widely used in the femur mechanical analysis, and this simplified consideration was shown to be adequate in hip reconstruction simulation [44]. Last, the spatial resolution of CT data used in this study was 3.0 mm, which is sufficient to reconstruct the bone geometry and determine the bone density, but not high enough to describe bone microstructure. Therefore, we used the reported data to validate the simulated L-T ratio in the presented study.

The bone material algorithm could be enhanced through this validation study by assigning optimal parameters to the model for minimizing the discrepancy between the simulated and real results. For example, the reference stimulus may vary among multiple regions [43, 45]; however, it is hard to choose appropriate reference stimulus value for individual regions without quantified standards. This validation study suggests an approach to optimize the reference stimulus: the real bone density determined by CT data in each element is chosen as the target value; the reference stimulus is adjusted repeatedly to minimize the gap between simulated density and real density, from which the optimal value of reference stimulus in each element can be determined. In the presented study, the same value of reference stimulus (e.g., 0.012) was assigned to every element, which results in overestimated bone density in the inferomedial and underestimated bone density in the superolateral region. Therefore, by assigning larger value of reference stimulus to the inferomedial and smaller value to the superolateral, one can improve the bone remodelling simulation accuracy. Besides, the bone remodelling algorithm is believed to be nonlinear rather than linear [18]; the remodelling coefficient and the order of nonlinear remodelling algorithm can be optimized by fitting the simulated data with real data via least squares method. Taken together, the suggested approach of integrating actual data with simulation results, along with the nonlinear bone material algorithm, will improve the bone remodelling prediction for future study.

The methods evaluating “error-driven” algorithms in the presented study are applicable to other bone material algorithms such as “reaction-diffusion” and “damage-repair” models mentioned above. Although used not as widely as the “error-driven” algorithm, these models integrate more biological mechanism into the material algorithm; thus, it could assist understanding how the bone cells participate in bone remodelling. The “reaction-diffusion” and “damage-repair” model has produced well-known features of the osteoporosis induced by imbalanced bone formation and resorption or by stress yielding after total hip replacement. These models, if improved by integrating CT data, may produce more accurate and realistic predicting results.

## 5. Conclusion

To conclude, this validation study suggests a role for bone material algorithm in prediction of bone amount and tissue material orientations in abnormal mechanical environment, such as during weightlessness or following total hip replacement. Furthermore, it has promise in the optimization of implant design for reducing the stress shielding and preventing fracture and is helpful in developing countermeasures against bone loss due to weightlessness. Besides, this study, through quantifying bone amount and trabecula structure, provides general methods for evaluation of the bone material algorithms. These methods will help investigate the roles of individual factors (e.g., force and load cycles) in bone remodelling by integrating the involved biological mechanisms and can enhance bone remodelling model by using optimal parameters to gap the discrepancy between the simulation and real data.
